# Surface Modification Strategy for Enhanced NO_2_ Capture in Metal–Organic Frameworks

**DOI:** 10.3390/molecules27113448

**Published:** 2022-05-26

**Authors:** Dionysios Raptis, Charalampos Livas, George Stavroglou, Rafaela Maria Giappa, Emmanuel Tylianakis, Taxiarchis Stergiannakos, George E. Froudakis

**Affiliations:** 1 Department of Chemistry, University of Crete, Voutes Campus, GR-71003 Heraklion, Crete, Greece; chemp1100@edu.chemistry.uoc.gr (D.R.); chemp1085@edu.chemistry.uoc.gr (C.L.); chemp1101@edu.chemistry.uoc.gr (G.S.); chemp956@edu.chemistry.uoc.gr (R.M.G.); sterg_t@chemistry.uoc.gr (T.S.); 2 Department of Materials Science and Technology, University of Crete, Voutes Campus, GR-71003 Heraklion, Crete, Greece; tilman@materials.uoc.gr

**Keywords:** metal–organic frameworks (MOFs), nitrogen dioxide (NO_2_), adsorption, density functional theory (DFT), grand canonical Monte Carlo (GCMC), functional group (FG)

## Abstract

The interaction strength of nitrogen dioxide (NO_2_) with a set of 43 functionalized benzene molecules was investigated by performing density functional theory (DFT) calculations. The functional groups under study were strategically selected as potential modifications of the organic linker of existing metal–organic frameworks (MOFs) in order to enhance their uptake of NO_2_ molecules. Among the functional groups considered, the highest interaction energy with NO_2_ (5.4 kcal/mol) was found for phenyl hydrogen sulfate (-OSO_3_H) at the RI-DSD-BLYP/def2-TZVPP level of theory—an interaction almost three times larger than the corresponding binding energy for non-functionalized benzene (2.0 kcal/mol). The groups with the strongest NO_2_ interactions (-OSO_3_H, -PO_3_H_2_, -OPO_3_H_2_) were selected for functionalizing the linker of IRMOF-8 and investigating the trend in their NO_2_ uptake capacities with grand canonical Monte Carlo (GCMC) simulations at ambient temperature for a wide pressure range. The predicted isotherms show a profound enhancement of the NO_2_ uptake with the introduction of the strongly-binding functional groups in the framework, rendering them promising modification candidates for improving the NO_2_ uptake performance not only in MOFs but also in various other porous materials.

## 1. Introduction

Nitrogen dioxide (NO_2_) belongs to a group of highly reactive gases known as nitrogen oxides (NOx) and primarily gets in the air from the burning of fuels [1]. The NO_2_ generated by the exhaust gases of the industries as well as by our private cars become an important air pollutant, the toxicity of which has an impact on the environment and human health [2]. NO_2_ is formed in the combustion processes of heating systems. The main pollutant emitted directly from hydrocarbon combustion is nitric oxide (NO), along with a small proportion of nitrogen dioxide (NO_2_). Nitrogen oxide is oxidized by ozone (O_3_) in the atmosphere, on a 10 min time scale, to give NO_2_ [3]. According to the EPA (Environmental Protection Agency), when NO_2_ is present in the air, it can be harmful to human health due to the irritation created in the airways of the human respiratory system [4]. In addition, NO_2_ and other NO_x_ molecules can interact with water, oxygen, and other chemicals in the atmosphere to form acid rain [5,6], whose harmful ecological effects are detrimental and most prominent in aquatic environments. Due to the serious impacts of nitrogen pollution [2], there are several separation methods of NO_2_ from industrial gas mixtures [7] and various adsorbents which have been used for the removal of NO_2_; some of them including zeolites [8], wood-based activated carbon [9], graphite oxides and iron composites [10]. Zhu et al., in an attempt to adsorb NO_2_ in N-doping activated carbon, observed that N-doping leads to easier adsorption of NO_2_ molecules, thus increasing NO_2_ physisorption energies [11]. After theoretically investigating NO_2_ adsorption on the surface of a silicon carbide (SiC) nanotube, Iranimanesh et al. proposed it for NO_2_ gas pollutant sensing and removal [12]. Lately, the separation of NO_2_ from industrial gas mixtures through its adsorption has been clearly identified as a possible application of metal–organic frameworks (MOFs) [13,14].

In the last two decades, there has been a rapid growth of metal–organic frameworks (MOFs) in the field of porous materials, and their applications vary from adsorption and separation of gases [15] to catalysis [16] and drug delivery [17]. MOFs are crystalline materials with extremely high porosity (up to 90% free volume) and a huge internal surface area extending beyond 6000 m^2^/g [18]. Due to these properties and considering the plethora of both inorganic and organic building blocks, MOFs have attracted great research interest as high-capacity adsorbents to meet various separation demands [18,19,20]. One of the important structure-to-property flexibilities of the MOF structures is the potential of their organic linkers to be modified with the incorporation of various functional groups in order to tune their interaction with selected molecules [18].

Several previous studies have shown that the introduction of functional groups into MOFs leads to enhanced gas uptake performance. In the work of Frysali et al. [21], the introduction of a sulfate anion in the phenyl ring has the highest interaction energy (−5.4 kcal/mol) with CO_2_, a value almost two times larger than the corresponding binding energy for benzene (−2.9 kcal/mol). Klontzas et al. [22] showed that the gravimetric capacity of the Li modified IRMOF-8 was calculated to 10 wt% at 77 K and 100 bar, while the corresponding values show great promise also at room temperature with an uptake of 4.5 wt%, performances significantly enhanced with respect to the unmodified counterpart (up to three times stronger). For NO_2_, the theoretical studies made by Fioretos et al. [23] have shown that NO_2_ interacts stronger with functionalized benzenes such as aniline, phenol, and toluene (with binding energies of −2.26 kcal/mol, −1.72 kcal/mol and −2.02 kcal/mol respectively) than with benzene (−1.67 kcal/mol). The amino (-NH_2_) substituent can be particularly beneficial as the interaction of strongly polar molecules, such as NO_2_, with the amino-substituted aromatic rings is characterized by the contribution of electrostatic dipole–dipole forces resulting in enhanced adsorption. 

Taking into account that many MOF frameworks have a phenyl group in their organic linker, together with the effectiveness of the organic linker functionalization strategy for tuning their interaction with guest molecules, in this work, we investigate the interaction of NO_2_ molecules with a series of 43 carefully selected benzene molecules by means of density functional theory (DFT) calculations. The selection of the functional groups was based on chemical intuition and findings of previous similar studies [22,24,25,26,27]. Subsequently, in order to verify the effectiveness of the functionalization on the enhancement of NO_2_ uptake in MOFs, grand canonical Monte Carlo (GCMC) simulations were performed for the IRMOF-8, modified by the three functional groups that showed the strongest interaction with NO_2_.

## 2. Computational Methods

### 2.1. Density Functional Theory

To investigate the interaction of NO_2_ molecules with the organic linkers of MOFs, we start with the simplified model of a benzene ring. A large set of 43 functional groups was examined for their binding strength towards the NO_2_ molecule. The conformations of the functionalized benzene molecules were optimized using Gershom Martin’s double-hybrid density functional DSD BLYP in the resolution of identity (RI) approximation [28] along with the def2-TZVPP basis set and with the corresponding auxiliary basis set for the RI approximation [29,30,31], including the D3BJ (Becke–Johnson damping version) empirical correction term for the dispersion interactions as proposed by Grimme [32,33,34,35]. All geometries were optimized without any symmetry constraints, and the optimized minimum-energy structures were verified as stationary points on the potential energy surface by performing numerical harmonic vibrational frequency calculations. All calculations were performed with the Orca 4.2 program package [36,37]. The dimer energies were corrected for the basis set superposition error (BSSE) using the counterpoise (CP) method as proposed by Boys and Bernardi [38].

The electron density redistribution was calculated as the difference between the electron density of the functionalized benzene–NO_2_ (ΔD) complex and the electron densities of the functionalized benzene (D(functionalized benzene)) and NO_2_ (D(NO_2_)) molecule according to the formula:

ΔD = D(functionalized benzene-NO_2_) − D(functionalized benzene) − D(NO_2_) (1)

All electron densities were calculated at the RI-DSD-BLYP/def2-TZVPP level of theory. Mathematical operations on the electron densities along with the visualization of the electron density difference, were done using gOpenMol graphics program [39,40]. 

### 2.2. Grand Canonical Monte Carlo

**Figure 1:**
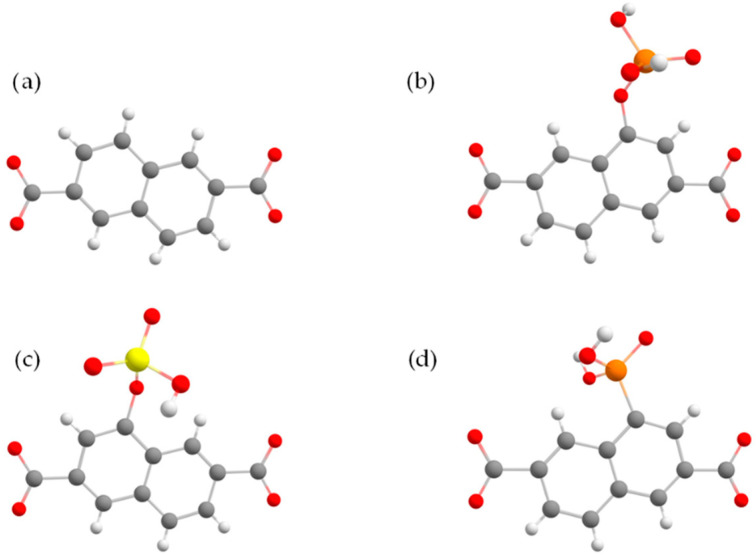
The functionalized linker of IRMOF-8 considered in the GCMC simulations; the original IRMOF-8 linker (**a**), the -OPO3H2 (**b**), -OSO3H (**c**), and -PO3H2 (**d**) functionalized linker. Carbon, hydrogen, oxygen, sulfur, and phosphorus atoms are depicted as gray, white, red, yellow, and orange spheres, respectively.

To verify the effectiveness of the strongest interacting functional group candidates obtained from the DFT calculations to enhance the NO_2_ uptake in MOFs, we employed Monte Carlo simulations in the grand canonical ensemble. IRMOF-8 was selected as the platform for the uptake calculation and was functionalized with the best performing functional groups, as shown in Figure 1.

The ΝO_2_ adsorption was calculated for a pressure range up to 1.2 bar at 298 K. Fugacity coefficients for the different thermodynamic states were defined using the Penge–Robinson equation of state [41]. The framework coordinates were taken from the crystallographic data [42] and a cubic periodic box of size 30.1 × 30.1 × 30.1 Å^3^ was used for all the frameworks, functionalized or parent. Simulations were performed in supercells incorporating enough repeat units such that all edge lengths were greater than 25.6 Å, i.e., twice the Lennard-Jones (LJ) cut-off radius. For each simulation point, 50,000 cycles were performed for system equilibration, followed by additional 100,000 cycles for sampling over the ensemble averages. For the description of the interactions between the IRMOF-8 and the NO_2_ atoms, LJ + Coulomb potentials were used and each atom of the host or the guest was treated explicitly [43]. The framework of IRMOF-8 was kept rigid during the simulations, while NO_2_ molecules were allowed to translate and rotate. Nitrogen dioxides were treated as three rigid center molecules with bond lengths between nitrogen and oxygen atoms held fixed at 1.19 Å. For the electrostatic interactions between NO_2_ molecules and the host material, point charges equal to qO = −0.073 and qN = 0.146 were placed at oxygen and nitrogen sites of NO_2_, respectively. The framework atoms charges were defined by employing the CHELPG method [44]. For the van der Waals interactions, potential parameters according to COMPASS Force Field [45,46] model were used, with ε = 50.36 and σ = 3.24 Å for Nitrogen atom and ε = 62.51 and σ = 2.93 Å for the oxygen center.

For each MOF framework, the potential parameters were taken from the UFF force field [47], except for organic linker atoms that were treated separately. Lorenz–Berthelot mixing rules were used to describe the NO_2_−IRMOF-8 interactions. For the functionalized organic linkers, the UFF parameters were found to be inconsistent with DFT interactions. To correct this, the parameters of the classical potential were fitted to reproduce the quantum chemical data (SI). All GCMC calculations were carried out with the RASPA software package [48].

## 3. Results and Discussion

**Figure 2:**
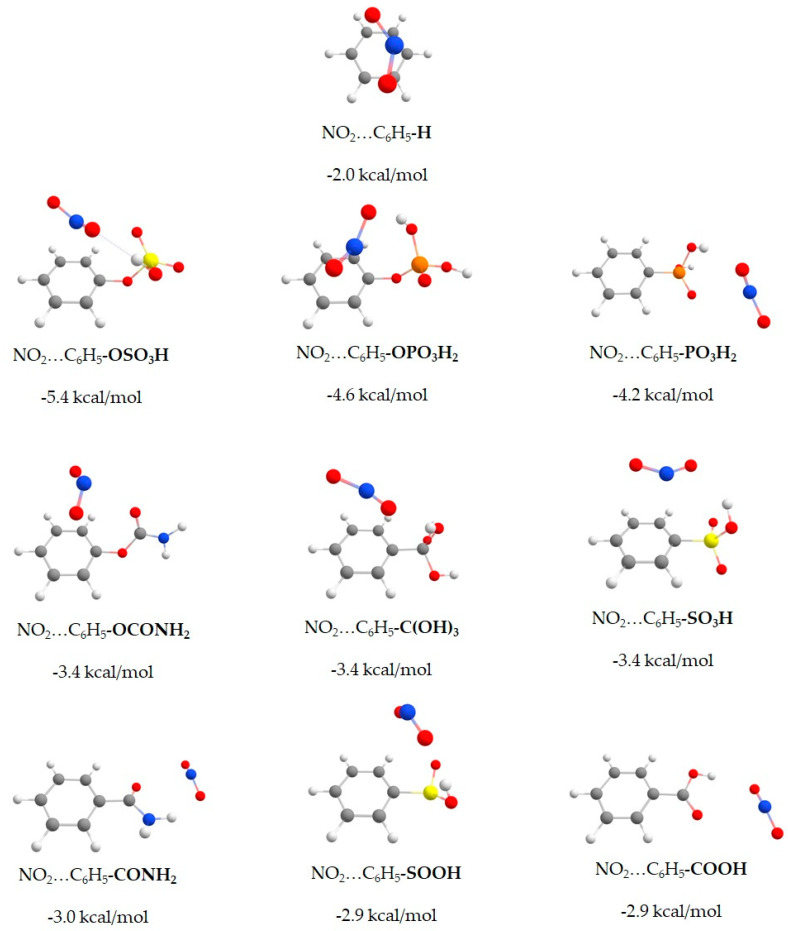
RI-DSD-BLYP D3(BJ) /def2-TZVPP optimized geometries of benzene and functionalized molecules interacting with NO_2_. Hydrogen, carbon, oxygen, nitrogen, lithium, sulfur, and phosphorus atoms are depicted as white, gray, red, blue, purple, yellow, and orange spheres, respectively.

The interaction of the NO_2_ molecule with the full set of the 43 strategically functionalized benzenes was calculated at the RI-DSD-BLYP/def2-TZVPP level of theory and can be seen in Table S1 of the SI section. In Table 1, we present the nine functional group candidates with the highest binding energy together with the non-functionalized benzene for comparison. In Figure 2, the DFT optimized dimer geometries of the NO_2_…C_6_H_5_-X systems are shown.

The highest interaction energy (5.4 kcal/mol) with NO_2_ was found for phenyl hydrogen sulfate (-OSO_3_H), which is almost three times stronger than the corresponding binding energy of the unfunctionalized benzene (2.0 kcal/mol). 

**Figure 3:**
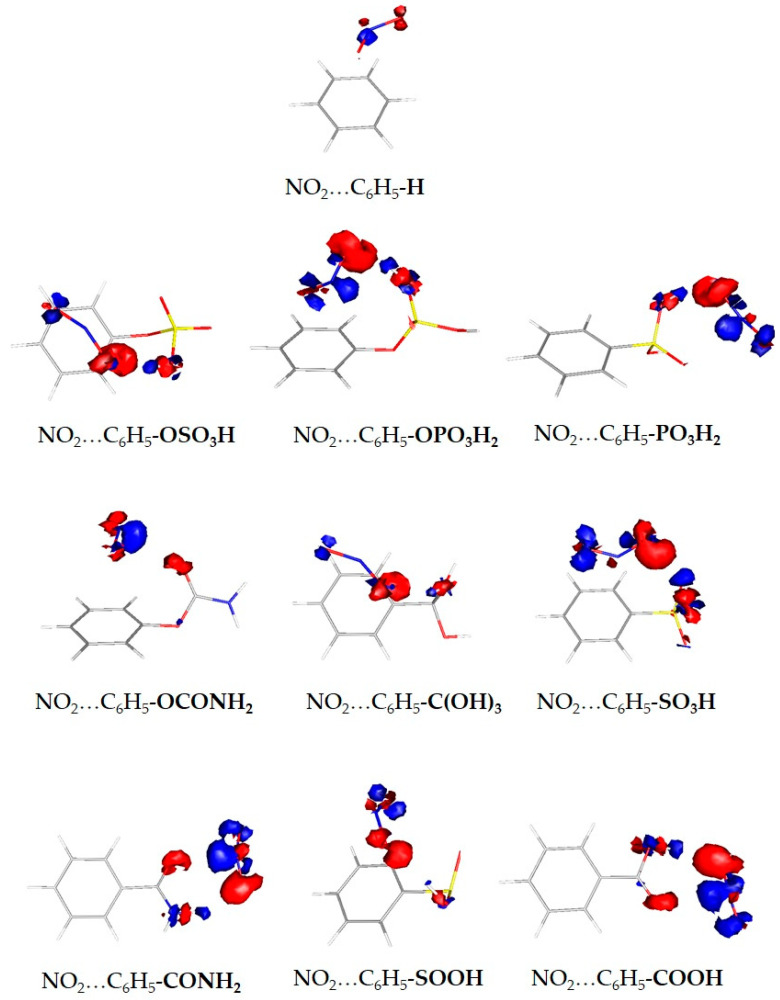
Electron density redistribution plots of the optimized geometries of the NO_2_…C_6_H_5_-X complexes. With red and blue the regions that gain and lose electron density upon the formation of the complex, respectively.

The energetically most favorable structures are characterized by a weak interaction between NO_2_ and organic linkers. More specifically, the acidic protons of the substituents, especially (-OSO_3_H, -OPO_3_H_2_, -PO_3_H_2_, -SOOH, -C(OH)_3_), tend to interact with NO_2_′s oxygen site with binding distances between 1.89 Å and 2.22 Å. The trend in the best binding energies is also confirmed by the electron density redistribution plots in Figure 3. Due to the electrostatic nature of NO_2_′s interactions, these plots can serve as a rule of thumb for predicting the most stable NO_2_ complexes with organic molecules. The red regions of the electron redistribution plots correspond to rich electron areas located around oxygen atoms, where the blue regions correspond to poor electron areas located mainly around hydrogen atoms. The electron-rich region of the nitrogen atom of NO_2_ interacts with the electron-poor regions of the functionalized benzene, and the electron-rich regions around oxygen atoms of NO_2_ interact with the electron-poor regions (around hydrogen atoms) of the functionalized benzenes.

**Figure 4:**
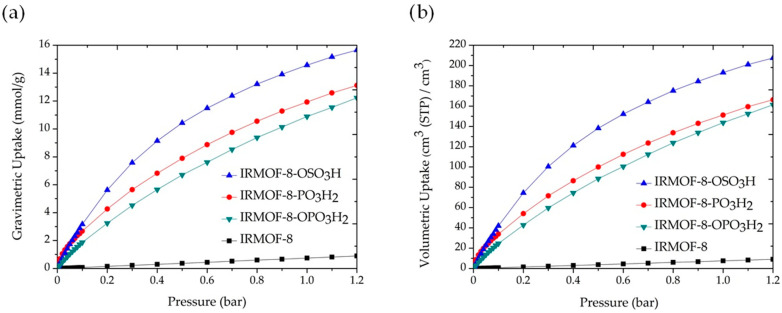
Gravimetric (**a**) and volumetric (**b**) NO_2_ uptake isotherms for IRMOF-8 and IRMOF-8-X (X: -OSO_3_H, -PO_3_H_2_, -OPO_3_H_2_) at 298 K.

To test the effect of the proposed surface modification strategy on the enhancement of the NO_2_ capture in MOF structures, we selected IRMOF-08 as the platform and modified its framework by adding one functional group per linker, as shown in Figure 1. We calculated the NO_2_ adsorption isotherms by performing GCMC simulations for the three functional groups (-OSO_3_H, -PO_3_H_2_, -OPO_3_H_2_) with the stronger interactions. From the excess volumetric and gravimetric isotherms at 298 K and pressures up to 1.2 bar (Figure 4a,b), there is a significant enhancement of the uptake due to functionalization. At 1.0 bar, the corresponding volumetric and gravimetric uptake for the IRMOF-8 with the OSO_3_H group was found at 193 cm^3^ (STP)/cm^3^ and 15 mmol/g, respectively, a value 12.8% larger than that of the unmodified IRMOF-8. The corresponding volumetric and gravimetric uptake for the unmodified IRMOF-8 was found at 7.5 cm^3^ (STP)/cm^3^ and 0.8 mmol/g. 

**Figure 5:**
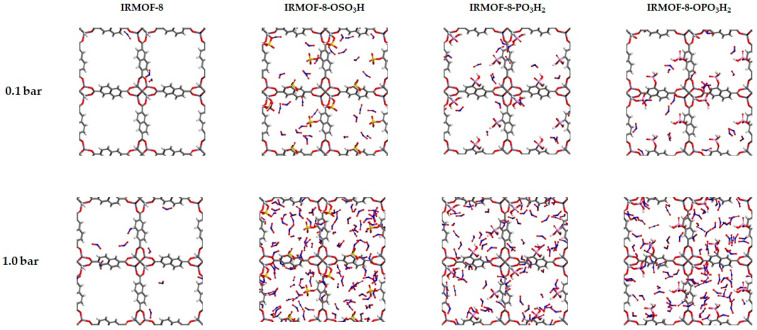
Snapshots for the IRMOF-8, IRMOF-8-X (X: -OSO3H, -PO_3_H_2_, -OPO_3_H_2_) from the GCMC at 298 K-0.1 bar and 298 K-1.0 bar.

Figure 5 shows representative snapshots taken at 0.1 and 1.0 bar for the parent and the three functionalized frameworks. In both pressures, the modified material hosts considerably more NO_2_ molecules than the parent structure due to the stronger binding sites introduced to the structure by functionalization. This is also verified by the fact that the NO_2_ molecules are located closer to the functional groups. 

## 4. Conclusions

In this work, we studied by means of density functional theory the interaction of a chemically diverse set of 43 functionalized benzenes with the NO_2_ molecule. 

The highest interaction energy with NO_2_ (5.4 kcal/mol at the RI-DSD-BLYP/def2-TZVPP level of theory) was found for phenyl hydrogen sulfate (-OSO_3_H)—an interaction almost three times larger than the corresponding binding energy for non-functionalized benzene (2.0 kcal/mol). 

The groups with the highest NO_2_ binding (-OSO_3_H, -PO_3_H_2_, -OPO_3_H_2_) were selected for functionalizing the linker of IRMOF-8 and investigating the trend in their NO_2_ uptake capacities with grand canonical Monte Carlo (GCMC) simulations. GCMC simulations showed a clear enhancement of the NO_2_ uptake both gravimetrically and volumetrically at 298 K and pressures up to 1.2 bar for the functionalized MOF, an enhancement even more pronounced at low pressures; at 0.1 bar, the volumetric uptake becomes 40 or 60 or 110 times larger than the unmodified IRMOF-08 by introducing -OPO_3_H_2_, -OSO_3_H, -PO_3_H_2_ functional groups, respectively. Based on this significant enhancement, we propose our surface functionalization as a general strategy for improving the NO_2_ adsorption uptake not only in MOFs, but also in various other porous materials. Our theoretical results can serve as high accuracy reference calculations and guide synthesis towards materials with high NO_2_ adsorption capacity. 

## Figures and Tables

**Table 1:** Binding energies in kcal/mol of the NO_2_…C_6_H_5_-X systems, calculated at the RI-DSD-BLYP D3(BJ)/def2-TZVPP level of theory and percentage of binding energy enhancement with respect to the introduction of the unfunctionalized benzene. All interaction energies are corrected for the BSSE by the full counterpoise method [39].

System	Binding Energy (kcal/mol)	Binding Energy Enhancement (%)
NO_2_…C_6_H_5_-**OSO_3_H**	−5.4	170%
NO_2_…C_6_H_5_-**OPO_3_H_2_**	−4.6	131%
NO_2_…C_6_H_5_-**PO_3_H_2_**	−4.2	110%
NO_2_…C_6_H_5_-**OCONH_2_**	−3.4	70%
NO_2_…C_6_H_5_-**C(OH)_3_**	−3.4	70%
NO_2_…C_6_H_5_-**SO_3_H**	−3.4	70%
NO_2_…C_6_H_5_-**CONH_2_**	−3.0	50%
NO_2_…C_6_H_5_-**SOOH**	−2.9	44%
NO_2_…C_6_H_5_-**COOH**	−2.9	44%
NO_2_…C_6_H_5_-**H**	−2.0	0%

## Data Availability

The data presented in this study are available in supplementary material.

## Supplementary Materials

The following supporting information can be downloaded at: https://www.mdpi.com/article/10.3390/molecules27113448/s1, Table S1: Sorted binding energies (kcal/mol) of the NO_2_…C_6_H_5_-X systems under study, calculated at the RI-DSD-BLYP D3(BJ)/def2-TZVPP level of theory. All interaction energy values have been corrected for the basis set superposition error (BSSE) by the full counterpoise method [38]. Percentage of binding energy enhancement with the introduction of the FG compared to benzene; Figure S1: Global minima geometries and binding energy values (in kcal/mol) of all the systems in this study; Figure S2: Electron density redistribution plots of the optimized geometries of the NO_2_…C_6_H_5_-X complexes. With red and blue being the regions that gain and lose electron density upon the formation of the complex, respectively; Figure S3: Fitting of the (ε, σ) parameters of the UFF [47] potential on the QM data obtained from the ab-initio scan of NO_2_ over benzene; Figure S4: Fitting of the (ε, σ) parameters for the NO_2_…C_6_H_5_-OPO_3_H_2_ interaction; Figure S5: Fitting of the (ε, σ) parameters for the NO_2_…C_6_H_5_-OSO_3_H interaction; Figure S6: Fitting of the (ε, σ) parameters for the NO_2_…C_6_H_5_-PO_3_H_2_ interaction.
