# Supplementary material for: Surface Modification Strategy for Enhanced NO2 Capture in Metal–Organic Frameworks
Source: Molecules. 2022 May 26;27(11):3448. doi: 10.3390/molecules27113448 (PMC9182044; doi:10.3390/molecules27113448)
Supplement: Supplementary file 1 [file molecules-27-03448-s001.zip › molecules-1711563-supplementary.pdf]

Supporting Information for:

Surface Modification Strategy for Enhanced NO2 Capture in Metal–Organic Frameworks

Dionysios Raptis [1], Charalampos Livas [1], George Stavroglou [1], Rafaela Maria Giappa [1,2], Emmanuel Tylianakis [2], Taxiarchis Stergiannakos [1] and George E. Froudakis [1,*]

[1] Department of Chemistry, University of Crete, Voutes Campus, GR-71003 Heraklion Crete, Greece; chemp1100@edu.chemistry.uoc.gr (D.R.); chemp1085@edu.chemistry.uoc.gr (C.L.); chemp1101@edu.chemistry.uoc.gr (G.S.); chemp956@edu.chemistry.uoc.gr (R.M.G.); sterg_t@chemistry.uoc.gr (T.S.)

[2] Department of Materials Science and Technology, University of Crete, Voutes Campus, GR-71003 Heraklion Crete, Greece; tilman@materials.uoc.gr

Density Functional Theory

Table S1. Sorted binding energies (kcal/mol) of the $NO_2 \ldots C_6H_5$-X systems under study, calculated at the RI-DSD-BLYP D3(BJ) /def2-TZVPP level of theory. All interaction energy values have been corrected for the Basis Set Superposition Error (BSSE) by the full counterpoise method [38]. Percentage of binding energy enhancement with the introduction of the FG compared to benzene.

System	Binding Energy (kcal/mol)	Binding Energy Enhancement (%)
$NO_2 \ldots C_6H_5$- **OSO_3H**	-5.4	170%
$NO_2 \ldots C_6H_5$- **OPO_3H_2**	-4.6	131%
$NO_2 \ldots C_6H_5$-**PO_3H_2**	-4.2	110%
$NO_2 \ldots C_6H_5$-**$OCONH_2$**	-3.4	70%
$NO_2 \ldots C_6H_5$-**$C(OH)_3$**	-3.4	70%
$NO_2 \ldots C_6H_5$-**SO_3H**	-3.4	70%
$NO_2 \ldots C_6H_5$-**$CONH_2$**	-3.0	50%
$NO_2 \ldots C_6H_5$-**SOOH**	-2.9	44%
$NO_2 \ldots C_6H_5$-**COOH**	-2.9	44%
$NO_2 \ldots C_6H_5$-**CH_2OH**	-2.9	44%
$NO_2 \ldots C_6H_5$-**OOH**	-2.8	41%
$NO_2 \ldots C_6H_5$-**CNH_2NOH**	-2.8	41%
$NO_2 \ldots C_6H_5$-**CH_2N_3**	-2.7	36%
$NO_2 \ldots C_6H_5$-**$CONHNH_2$**	-2.7	36%
$NO_2 \ldots C_6H_5$-**$CONHCH_3$**	-2.7	36%
$NO_2 \ldots C_6H_5$-**SO_2NH_2**	-2.7	36%
$NO_2 \ldots C_6H_5$-**NCH_2**	-2.7	36%
$NO_2 \ldots C_6H_5$-**CH_2NH_2**	-2.6	31%
$NO_2 \ldots C_6H_5$-**NNH**	-2.6	31%
$NO_2 \ldots C_6H_5$-**CHNOH**	-2.5	26%
$NO_2 \ldots C_6H_5$-**$NHCOCH_3$**	-2.5	26%
$NO_2 \ldots C_6H_5$-**NH_2**	-2.4	21%
$NO_2 \ldots C_6H_5$-**O_2CH_2**	-2.4	21%
$NO_2 \ldots C_6H_5$- **OC_2H_5**	-2.3	16%
$NO_2 \ldots C_6H_5$-**CH_3**	-2.3	16%

NO_2…C_6H_5-**PH_2**	-2.3	16%
NO_2…C_6H_5-**$COOCH_3$**	-2.2	11%
NO_2…C_6H_5-**SO_2CH_3**	-2.2	11%
NO_2…C_6H_5-**CHNH**	-2.2	11%
NO_2…C_6H_5-**COOCHO**	-2.2	11%
NO_2…C_6H_5-**CHO**	-2.2	11%
NO_2…C_6H_5-**CF_3**	-2.1	6%
NO_2…C_6H_5-**SH**	-2.1	6%
NO_2…C_6H_5-**N_3**	-2.1	6%
NO_2…C_6H_5-**NCO**	-2.0	0%
NO_2…C_6H_5-**$CHCHNO_2$**	-2.0	0%
NO_2…C_6H_5-**H**	-2.0	0%
NO_2…C_6H_5-**NCS**	-1.9	-5%
NO_2…C_6H_5-**F**	-1.9	-5%
NO_2…C_6H_5-**CN**	-1.9	-5%
NO_2…C_6H_5-**SCN**	-1.9	-5%
NO_2…C_6H_5-**SO_2Cl**	-1.7	-15%
NO_2…C_6H_5-**NC**	-1.6	-20%
NO_2…C_6H_5-**OH**	-1.5	-25%

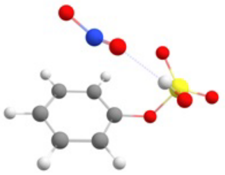

NO_2…C_6H_5-**OSO_3H**

-5.4 kcal/mol

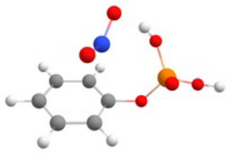

NO_2…C_6H_5-**OPO_3H_2**

-4.6 kcal/mol

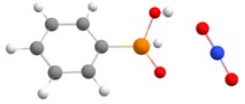

NO_2…C_6H_5-**PO_3H_2**

-4.2 kcal/mol

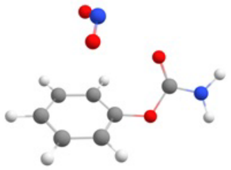

NO_2…C_6H_5-**$OCONH_2$**

-3.4 kcal/mol

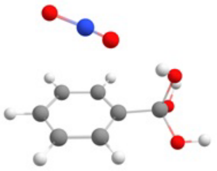

NO_2…C_6H_5-**$C(OH)_3$**

-3.4 kcal/mol

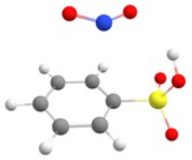

NO_2…C_6H_5-**SO_3H**

-3.4 kcal/mol

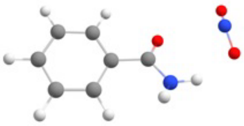

NO_2…C_6H_5-**$CONH_2$**

-3.0 kcal/mol

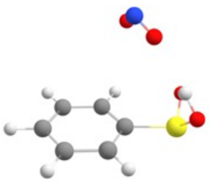

NO_2…C_6H_5-**SOOH**

-2.9 kcal/mol

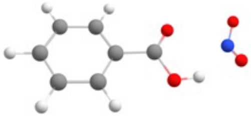

NO_2…C_6H_5-**COOH**

-2.9 kcal/mol

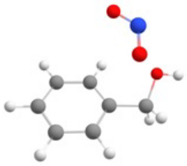

NO_2…C_6H_5-**CH_2OH**

-2.9 kcal/mol

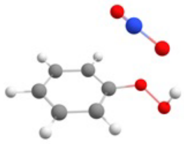

NO_2…C_6H_5-**OOH**

-2.8 kcal/mol

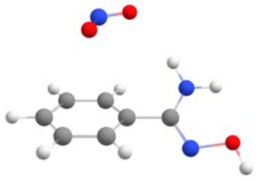

NO_2…C_6H_5-**CNH_2NOH**

-2.8 kcal/mol

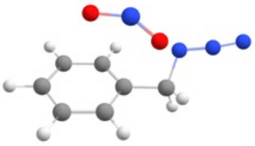

NO_2…C_6H_5-**CH_2N_3**

-2.7 kcal/mol

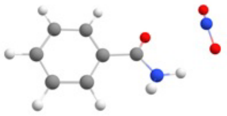

NO_2…C_6H_5-**$CONHNH_2$**

-2.7 kcal/mol

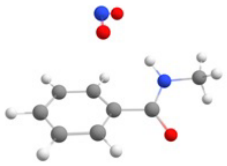

NO_2…C_6H_5-**$CONHCH_3$**

-2.7 kcal/mol

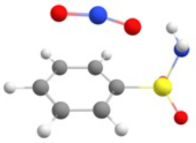

NO_2…C_6H_5-**SO_2NH_2**

-2.7 kcal/mol

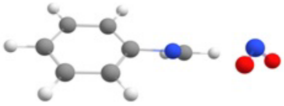

$NO_2 \ldots C_6H_5$-**NCH_2**

-2.7 kcal/mol

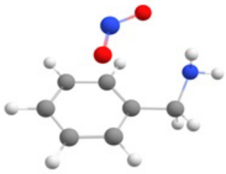

$NO_2 \ldots C_6H_5$-**CH_2NH_2**

-2.6 kcal/mol

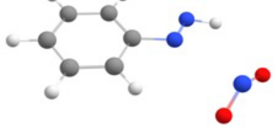

$NO_2 \ldots C_6H_5$-**NNH**

-2.6 kcal/mol

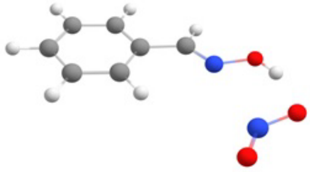

$NO_2 \ldots C_6H_5$-**CHNOH**

-2.5 kcal/mol

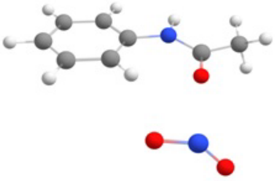

$NO_2 \ldots C_6H_5$-**$NHCOCH_3$**

-2.5 kcal/mol

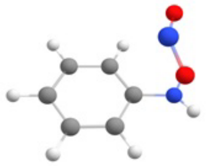

$NO_2 \ldots C_6H_5$-**NH_2**

-2.4 kcal/mol

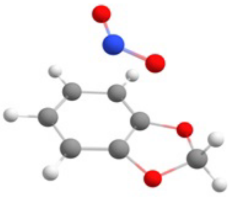

$NO_2 \ldots C_6H_5$-**O_2CH_2**

-2.4 kcal/mol

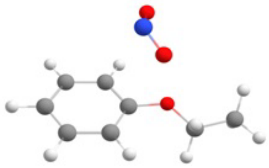

$NO_2 \ldots C_6H_5$-**OC_2H_5**

-2.3 kcal/mol

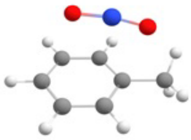

$NO_2 \ldots C_6H_5$-**CH_3**

-2.3 kcal/mol

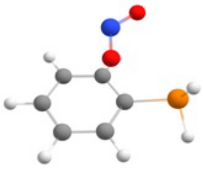

$NO_2 \ldots C_6H_5$-**PH_2**

-2.3 kcal/mol

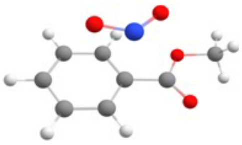

$NO_2 \ldots C_6H_5$-**$COOCH_3$**

-2.2 kcal/mol

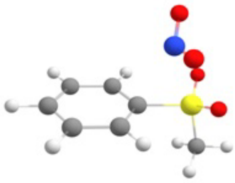

$NO_2 \ldots C_6H_5$-**SO_2CH_3**

-2.2 kcal/mol

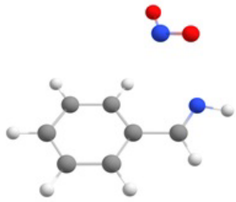

$NO_2 \ldots C_6H_5$-**CHNH**

-2.2 kcal/mol

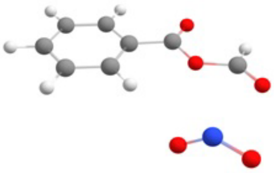

$NO_2 \ldots C_6H_5$-**COOCHO**

-2.2 kcal/mol

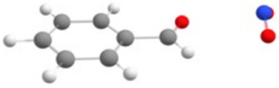

$NO_2 \ldots C_6H_5$-**CHO**

-2.2 kcal/mol

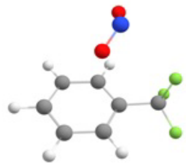

$NO_2 \ldots C_6H_5$-**CF_3**

-2.1 kcal/mol

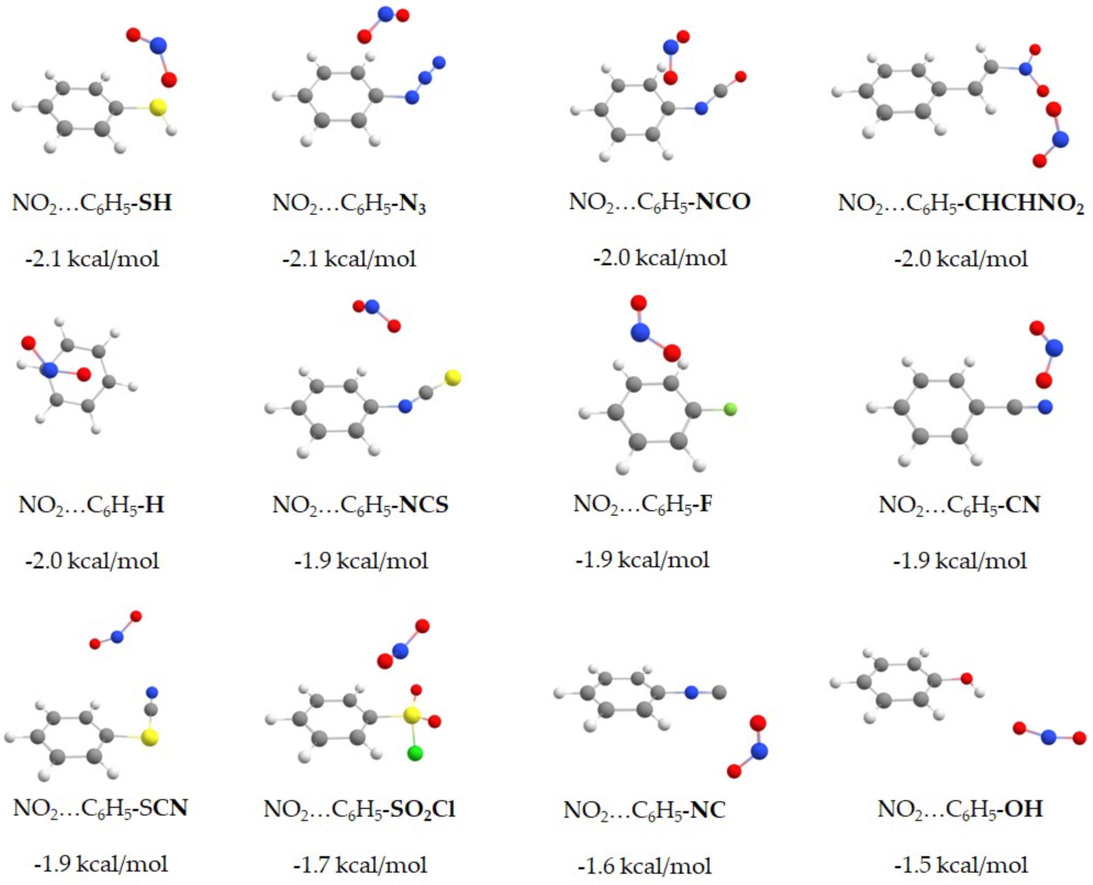

Figure S1. Global minima geometries and binding energy values (in kcal/mol) of all the systems in this study.

Densities were plotted with a contour value of 0.001 au by using gOpenMol [39,40] and are shown in Figure S2.

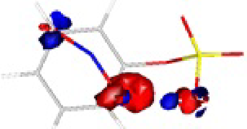

$NO_2 \ldots C_6H_5$-**OSO_3H**

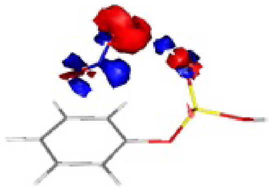

$NO_2 \ldots C_6H_5$-**OPO_3H_2**

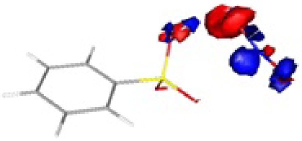

$NO_2 \ldots C_6H_5$-**PO_3H_2**

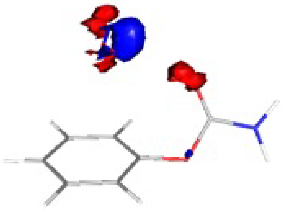

$NO_2 \ldots C_6H_5$-**$OCONH_2$**

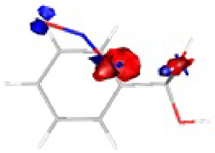

$NO_2 \ldots C_6H_5$-**$C(OH)_3$**

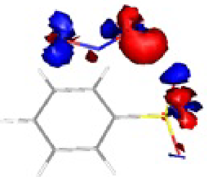

$NO_2 \ldots C_6H_5$-**SO_3H**

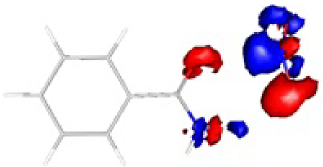

$NO_2 \ldots C_6H_5$-**$CONH_2$**

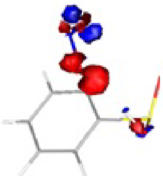

$NO_2 \ldots C_6H_5$-**SOOH**

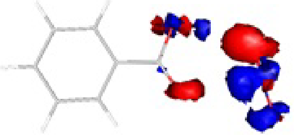

$NO_2 \ldots C_6H_5$-**COOH**

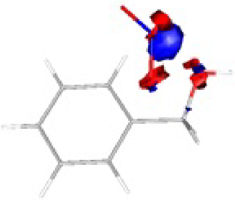

$NO_2 \ldots C_6H_5$-**CH_2OH**

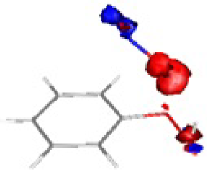

$NO_2 \ldots C_6H_5$-**OOH**

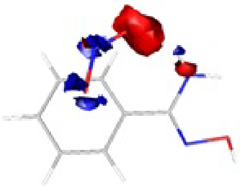

$NO_2 \ldots C_6H_5$-**CNH_2NOH**

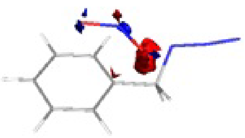

$NO_2 \ldots C_6H_5$-**CH_2N_3**

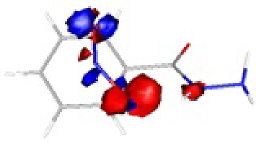

$NO_2 \ldots C_6H_5$-**$CONHNH_2$**

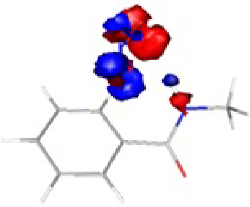

$NO_2 \ldots C_6H_5$-**$CONHCH_3$**

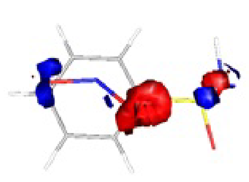

NO_2…C_6H_5.**SO_2NH_2**

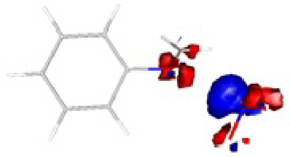

NO_2…C_6H_5.**NCH_2**

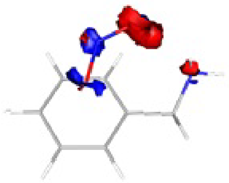

NO_2…C_6H_5.**CH_2NH_2**

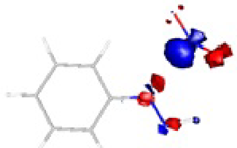

NO_2…C_6H_5-**NNH**

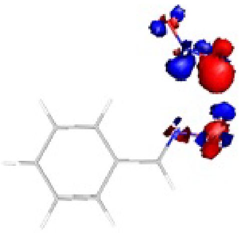

NO_2…C_6H_5-**CHNOH**

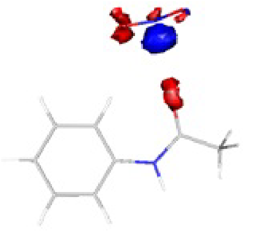

NO_2…C_6H_5-**$NHCOCH_3$**

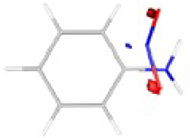

NO_2…C_6H_5-**NH_2**

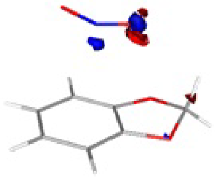

NO_2…C_6H_5-**O_2CH_2**

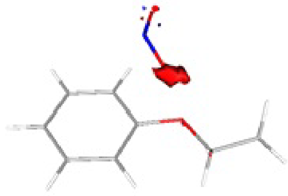

NO_2…C_6H_5-**OC_2H_5**

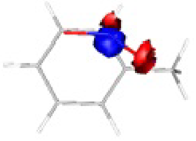

NO_2…C_6H_5-**CH_3**

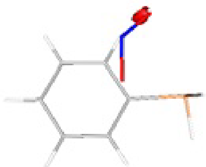

NO_2…C_6H_5-PH_2

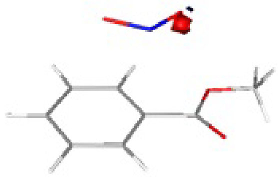

NO_2…C_6H_5-**$COOCH_3$**

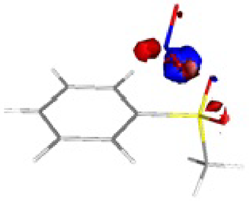

NO_2…C_6H_5-**SO_2CH_3**

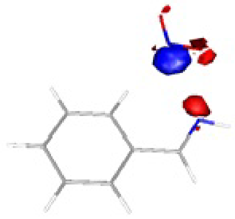

NO_2…C_6H_5-**CHNH**

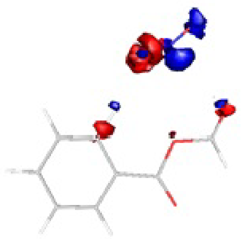

NO_2…C_6H_5-**COOCHO**

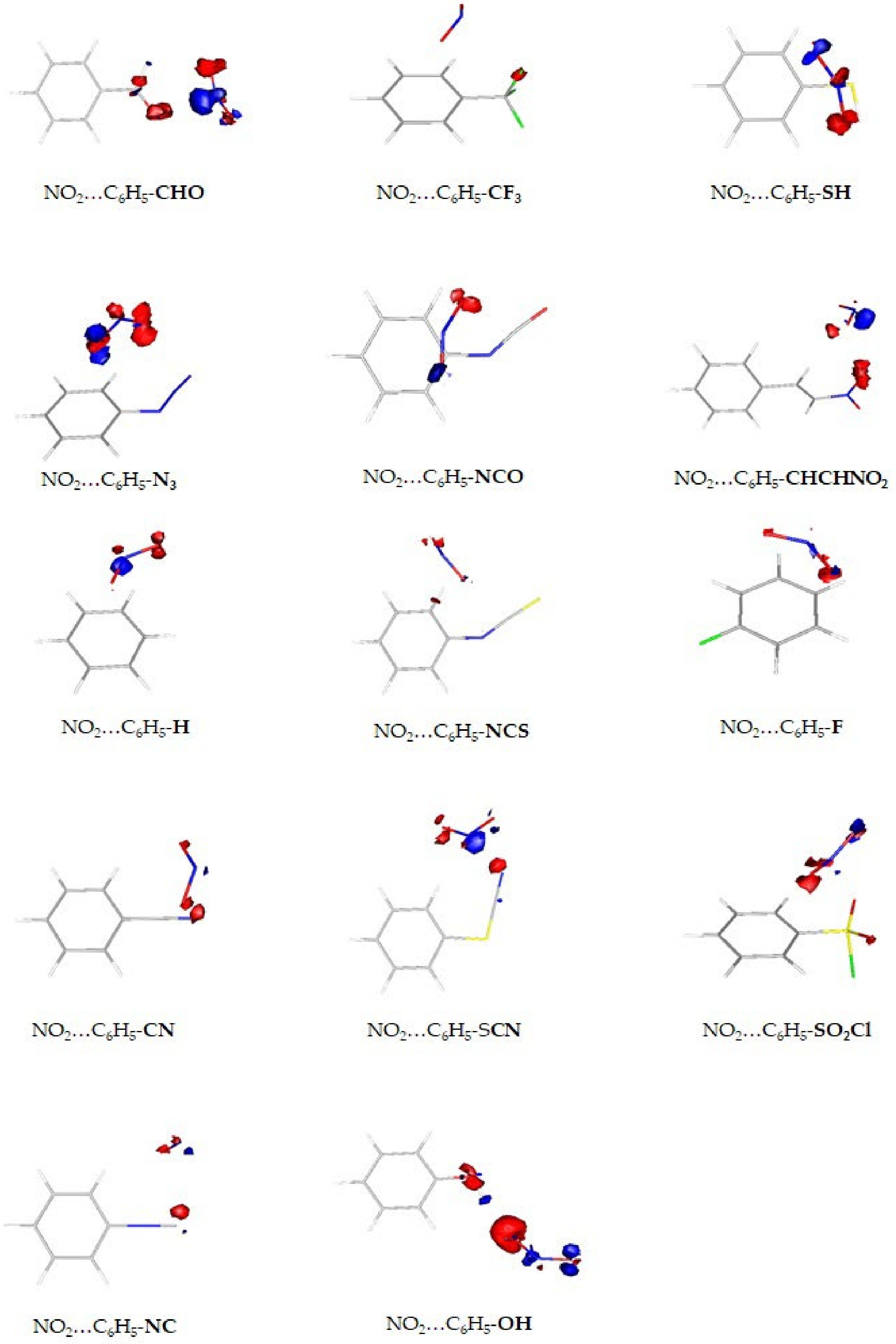

Figure S2. Electron density redistribution plots of the optimized geometries of the NO_2…C_6H_5-X complexes. With red and blue the regions that gain and lose electron density upon the formation of the complex, respectively.

Grand Canonical Monte Carlo

A rigid scan was performed at the RI-DSD-BLYP D3(BJ) /def2-TZVPP level for each functional group selected to determine the interaction energies between the NO_2 molecule and the C_6H_5-X.

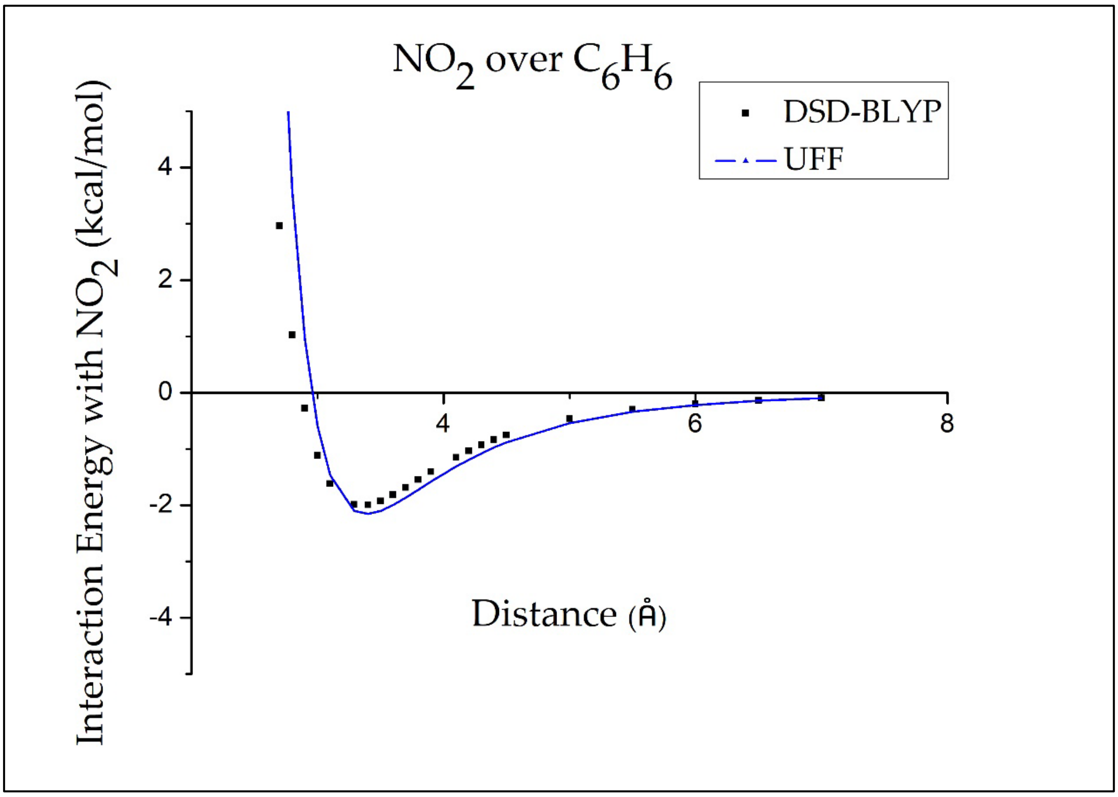

Figure S3. Fitting of the (ε, σ) parameters of the UFF [47] potential on the QM data obtained from the DFT scan of NO_2 over benzene.

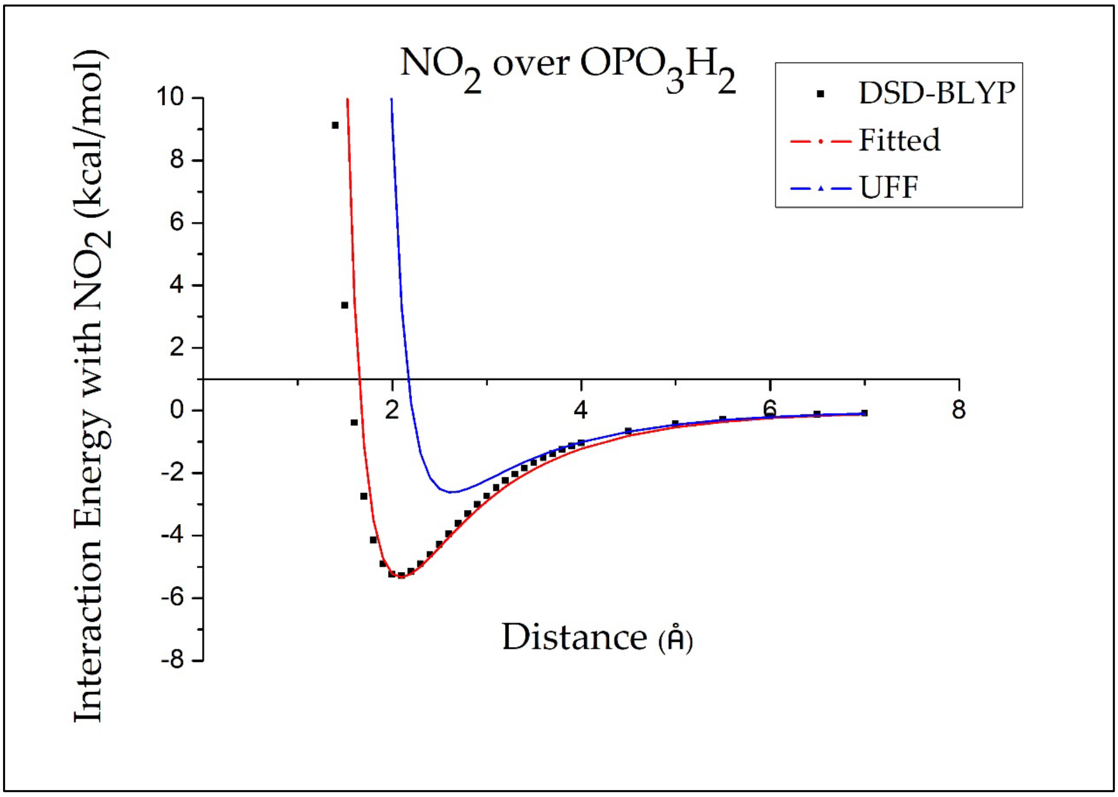

Figure S4. Fitting of the (ε, σ) parameters for the NO_2…C_6H_5-OPO_3H_2 interaction.

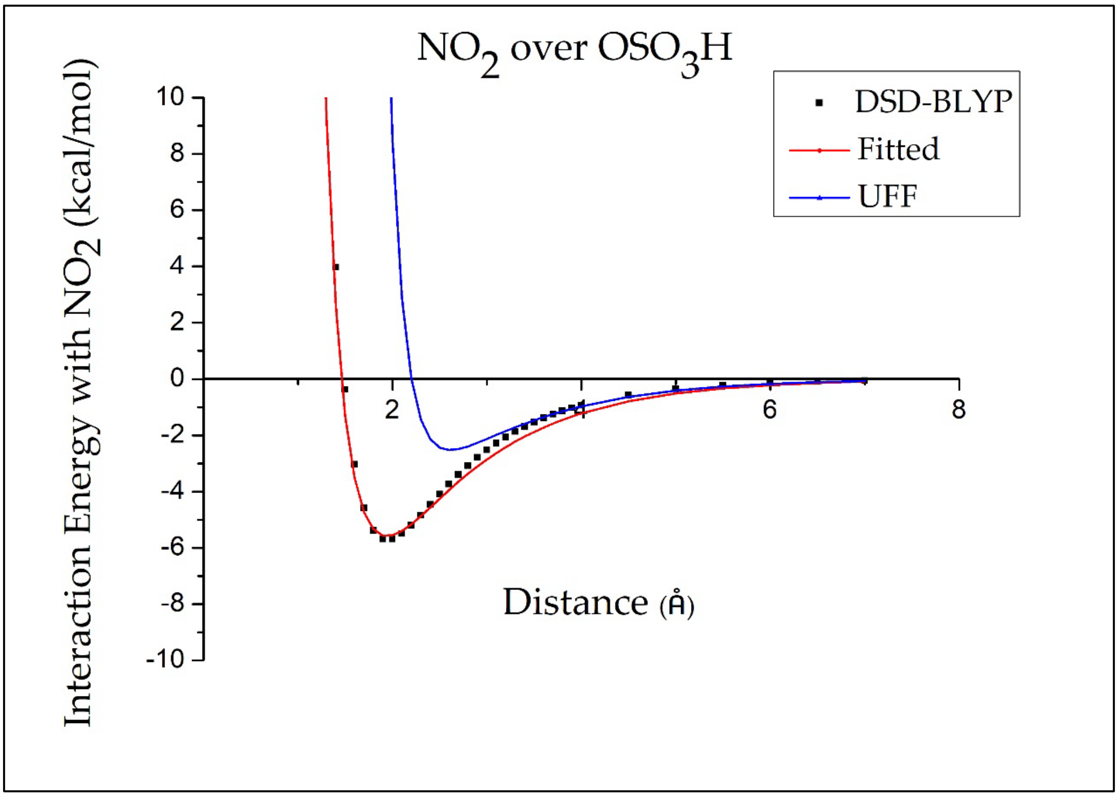

Figure S5. Fitting of the (ε, σ) parameters for the NO_2…C_6H_5-OSO_3H interaction.

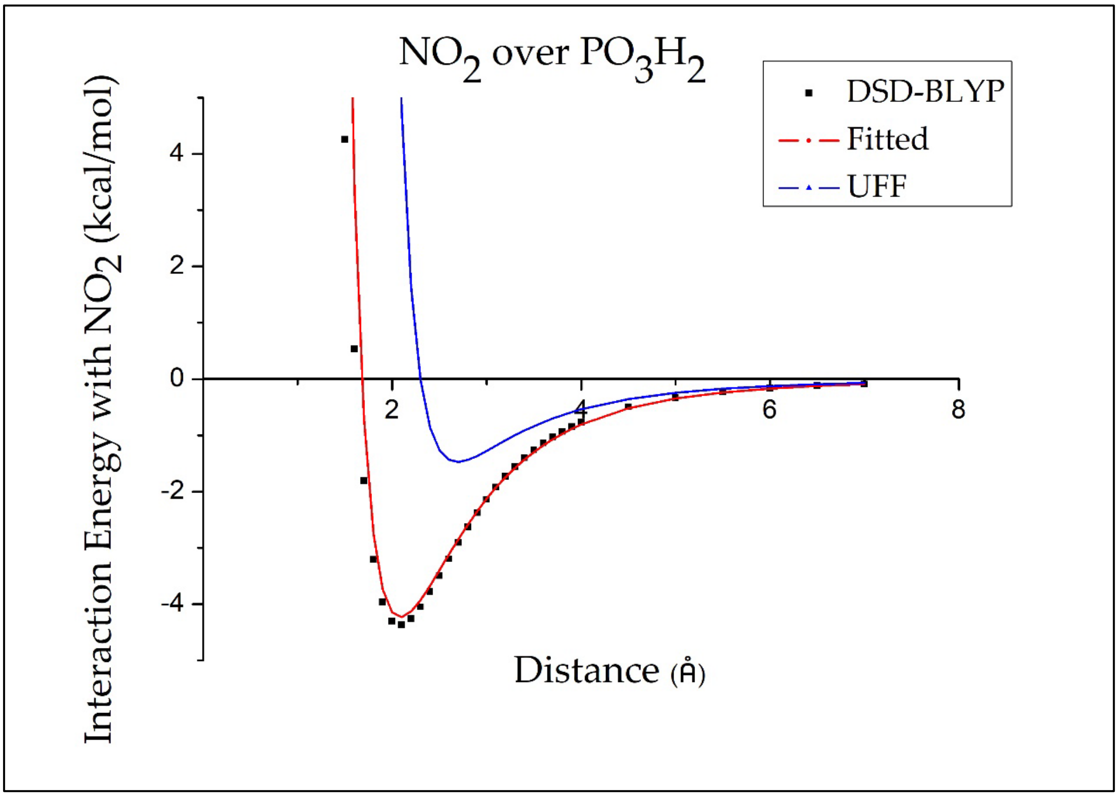

Figure S6. Fitting of the (ε, σ) parameters for the $NO_2 \ldots C_6H_5\text{-}PO_3H_2$ interaction.
